# A comparative study on the clinical efficacy of microplate trans-carpometacarpal joint fixation and non-trans-carpometacarpal joint fixation in treating fractures with dislocation or subluxation of the base of the fourth and fifth metacarpal bones

**DOI:** 10.1186/s13018-023-04225-2

**Published:** 2023-09-28

**Authors:** Liang Zhao

**Affiliations:** Department of Hand and Foot Surgery, Shunyi District Hospital, No. 3 Guangming South Street, Shunyi District, Beijing, 101300 China

**Keywords:** Carpometacarpal joint, Metacarpal base, Fracture and dislocation, Fracture fixation, Hand

## Abstract

**Background:**

This study aimed to compare the clinical efficacy of microplate trans-carpometacarpal joint fixation and non-trans-carpometacarpal joint fixation in treating fractures and dislocation or subluxation of the base of the fourth and fifth metacarpal bones.

**Method:**

From 2015 to 2021, 100 cases of metacarpal basal fractures with dislocation or subluxation were randomly divided into the trans-carpometacarpal joint fixation group (group A) and non-trans-carpometacarpal joint fixation group (group B). Group A (n = 50) comprised 44 males and 6 females, with an average age of 28.8 ± 6.1 y and an Orthopedic Trauma Association (OTA) fracture classification of type B1 (n = 29) or C1 (n = 21). Group B (n = 50) comprised 45 males and 5 females, with an average age of 28.9 ± 5.7 y and an OTA fracture classification of type B1 (n = 28) or C1 (n = 22). All patients were complicated with dislocation or subluxation. The surgery time, fracture healing time, postoperative handgrip strength, and total active motion (TAM) scores of the ring and little fingers were recorded and compared between the two groups. The clinical efficacy of patients was evaluated using scoring methods such as DASH (disabilities of the arm, shoulder and hand), visual analogue scale (VAS), and Mayo at 3, 6, and 12 months after surgery.

**Results:**

There was no significant difference in the general indexes, surgery time, or fracture healing time between the two groups (*P* > 0.05). There were no significant differences in handgrip strength and TAM scores of the ring and little fingers between the two groups at 3 and 12 months postoperatively (*P* > 0.05), but there were significant differences in these indexes 6 months postoperatively (*P* < 0.05). There were no significant differences in the DASH, VAS, and Mayo scores at 3 and 12 months postoperatively (*P* > 0.05), but there were significant differences between the two groups in the DASH and Mayo scores (*P* < 0.05) but not the VAS score (*P* > 0.05) 6 months postoperatively.

**Conclusion:**

In the treatment of fourth and fifth metacarpal basal fractures with dislocation or subluxation, both microplate transarticular fixation and non-transarticular fixation could achieve fracture fixation and healing, and each method had advantages and disadvantages. The clinically appropriate fixation method should be selected according to the experience of the surgeon and the degree and type of fracture and dislocation.

## Background

Basal fractures of the fourth and fifth metacarpal bones resulting from hand trauma are clinically common and are often complicated with dislocation or subluxation of the carpometacarpal joint. If the treatment method is not appropriate, this injury can easily lead to sequelae, such as joint stiffness, pain, weakness, or joint degeneration [1, 2]. There are many treatment methods available for such fractures, e.g., conservative plaster fixation, Kirschner wire fixation, an external fixation frame [3], and micro-steel plate internal fixation [4, 5]. Considering improvements in surgical technique, nursing, and surgical materials, as well as the needs of patients to return quickly to normal activities, open reduction, combined with microplate internal fixation, has become a safe and effective clinical method of treatment. Xu et al. [6] retrospectively analyzed that micro plate internal fixation can achieve good reduction, firm and reliable, but for comminuted fractures, it could not be satisfactorily fixed. Scohortinghuis [7] believed that when the metacarpal base was comminuted or combined with dorsal fracture of the hamate, it was a good choice to use the micro steel plate to fix across the carpometacarpal joint, which can support the open and close joint, especially for comminuted fractures, which can prevent the loss after fracture reduction. However, after fixation of the carpometacarpal joint, the range of motion was reduced, which affects the function of the hand [8]. However, there is no consensus about whether to use a microplate to directly fix a fracture or enable trans-carpometacarpal joint fixation after open fracture reduction.

There is no uniform consensus on the surgical method for such trauma. For this reason, 100 patients with metacarpal basal fracture dislocation or subluxation caused by hand trauma were randomly divided into two groups from 2015. They were treated with trans-carpometacarpal joint fixation and non-trans-carpometacarpal joint fixation, and their clinical efficacy was observed and followed up to provide basis for the selection of surgical methods for such patients in the future. Our hypothesis was that both fixation methods could achieve fracture dislocation healing, but there were still differences in the recovery process.

## Information and method

### Inclusion and exclusion criteria

Inclusion criteria: (1) Age > 14 years old, (2) Clear history of hand injury, (3) radiograph and computed tomography (CT) examination: fourth or (and) fifth metacarpal basal fracture, accompanied by dislocation or subluxation, Exclusion criteria: (1) Age ≤ 14 years old, (2) Radiograph and CT examination: Fourth or (and) fifth metacarpal basal fracture alone or only dislocation without fracture, (3) Patients with residual hand dysfunction caused by pathological fractures, cerebral thrombosis, or other diseases.

### General information

**Fig. 1:**
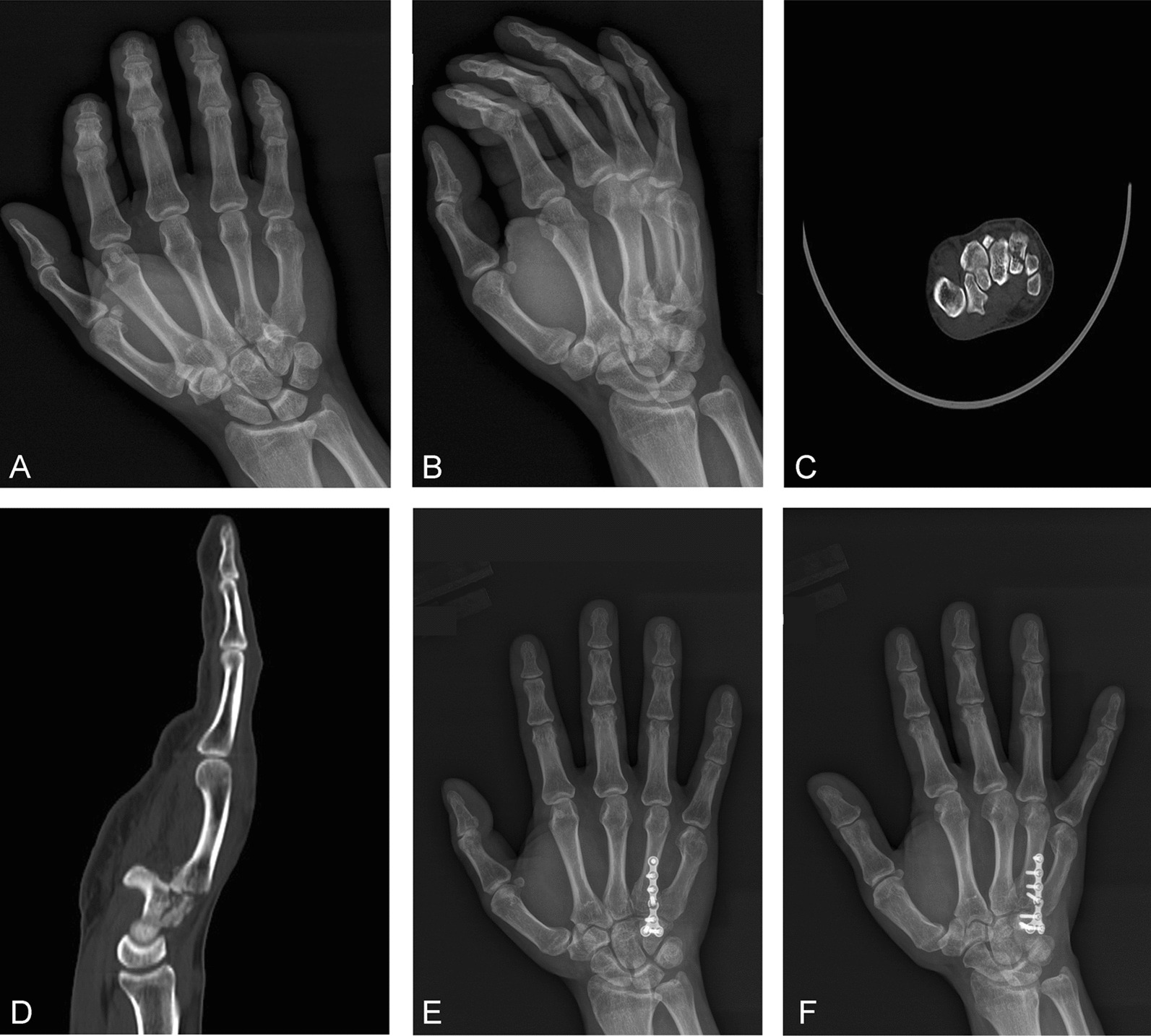
**A**, **B** The preoperative anteroposterior oblique films of the affected hand indicate that the fracture of the base of the fourth metacarpal bone is complicated with dislocation. **C**, **D** Preoperative computed tomography images of the affected hand indicate that there is dorsal dislocation of the fracture of the base of the fourth metacarpal bone and a hamate bone fracture. **E**, **F** Three months postoperatively, the posteroanterior and lateral oblique views on radiograph show that the transarticular steel plate is broken

**Fig. 2:**
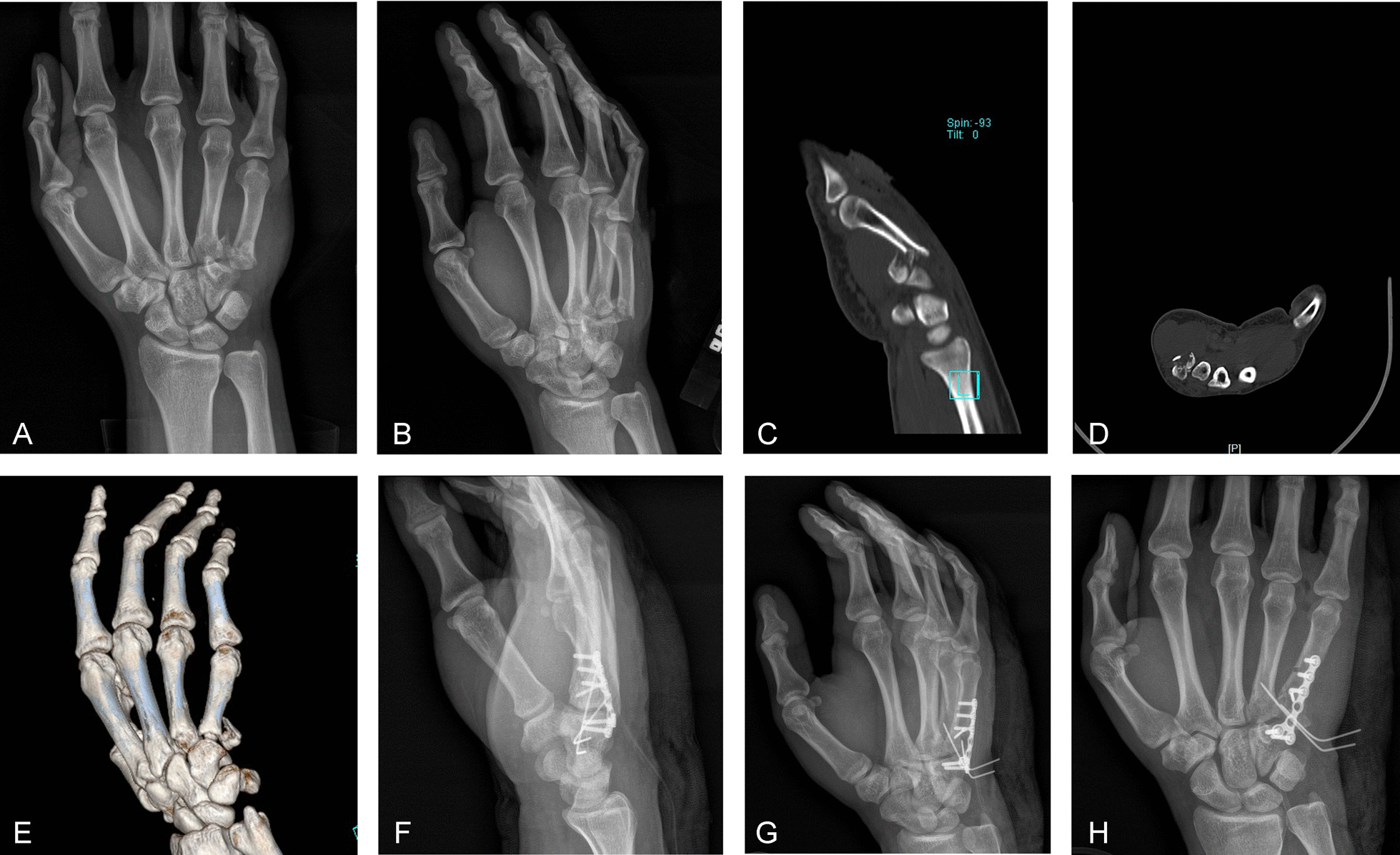
**A**, **B** The preoperative posteroanterior and lateral oblique views of the affected hand on radiograph indicate that there are comminuted fractures of the base of the fifth metacarpal bone, complicated with dislocation. **C**, **D**, **E** Preoperative computed tomography and three-dimensional reconstruction of the affected hand show a comminuted fracture of the base of the fifth metacarpal bone that is complicated with dislocation. **F**, **G**, and **H** The postoperative posteroanterior and lateral oblique views of the affected hand on radiograph show that a plate was used for transarticular fixation, and Kirschner wire was used to assist in the fixation of bone fragments

From January 2015 to December 2021, 100 patients with metacarpal basal fracture dislocation or subluxation who were treated in the Beijing Shunyi District Hospital were selected. All patients were randomly assigned using a random number table method and divided into the transarticular fixation group (Group A) and the non-transarticular fixation group (Group B). The group A comprised 50 patients, including 44 males and 6 females with an average age of 28.8 ± 6.1 y (18–54 y). The injuries in Group A included fracture of the fourth metacarpal base with dislocation or subluxation (n = 5), fracture of the fifth metacarpal base with dislocation or subluxation (n = 22), and fracture of the fourth and fifth metacarpal base with dislocation or subluxation (n = 23); 45 cases of injury involved the right hand and 5 cases of injury involved the left hand. The injuries were caused by falling (n = 10), boxing (n = 33), and traffic accidents (n = 7). The Orthopedic Trauma Association (OTA) [9] fracture classifications were B1 (n = 29) and C1 (n = 21), and all patients were complicated with dislocation or subluxation. All the injuries were closed fractures, including 9 cases complicated with a hamate fracture. In all patients, the posteroanterior and lateral oblique views of the hand were examined preoperatively via radiograph and computed tomography (CT). The typical images taken during surgery in group A are shown in Figs. 1 and 2.

**Fig. 3:**
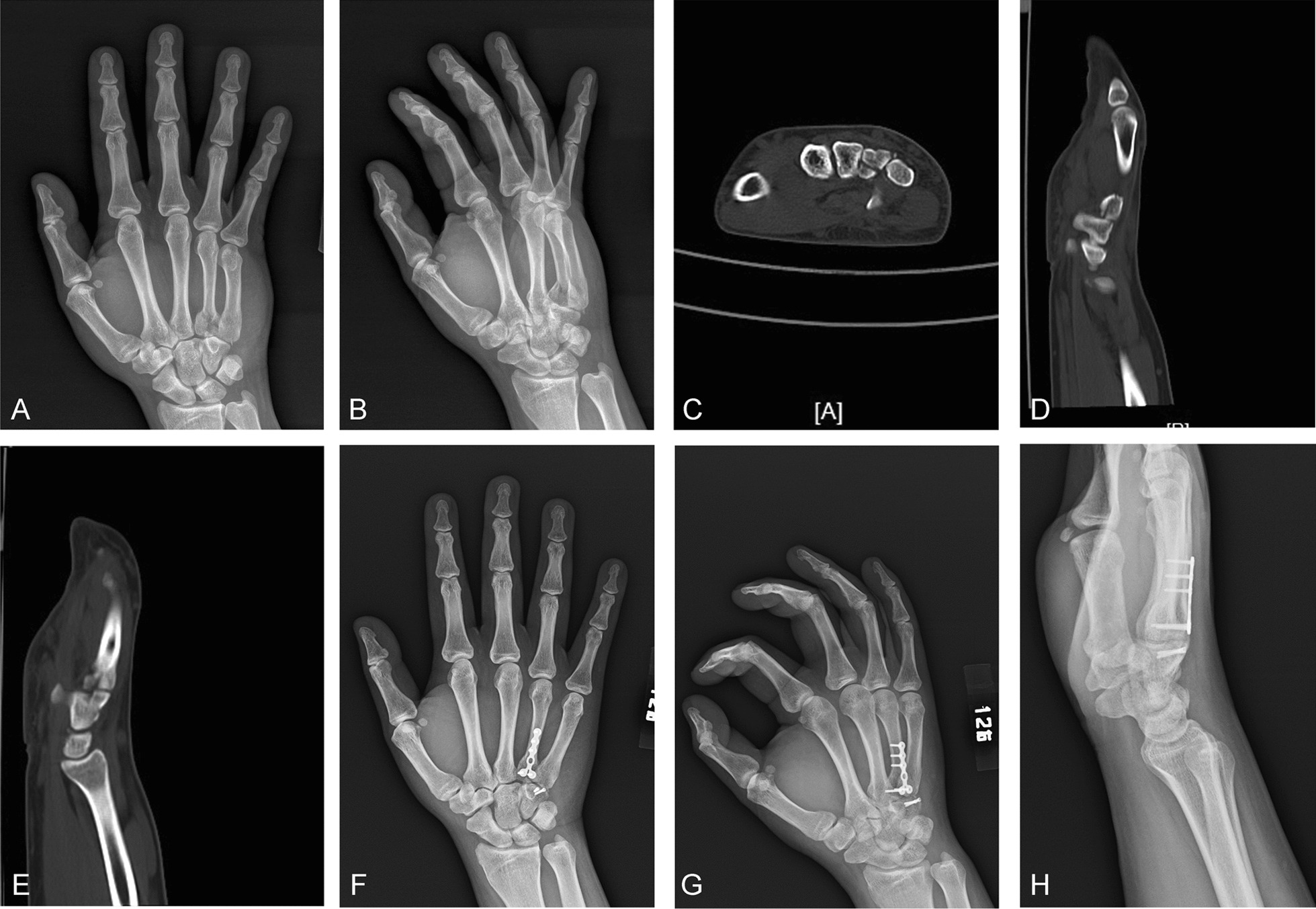
**A**, **B** The preoperative posteroanterior and lateral oblique views of the affected hand on radiograph indicate that the fracture of the base of the fourth metacarpal bone is complicated with dislocation. **C**, **D**, and **E** Preoperative computed tomography images of the affected hand indicate a dorsal dislocation of the fracture of the base of the fourth metacarpal bone and a hamate bone fracture. **F**, **G**, and **H** One-month postoperative posteroanterior and lateral oblique views on radiograph show that the microplate was not fixed across the joint, and the hamate bone fracture was fixed with two screws

**Fig. 4:**
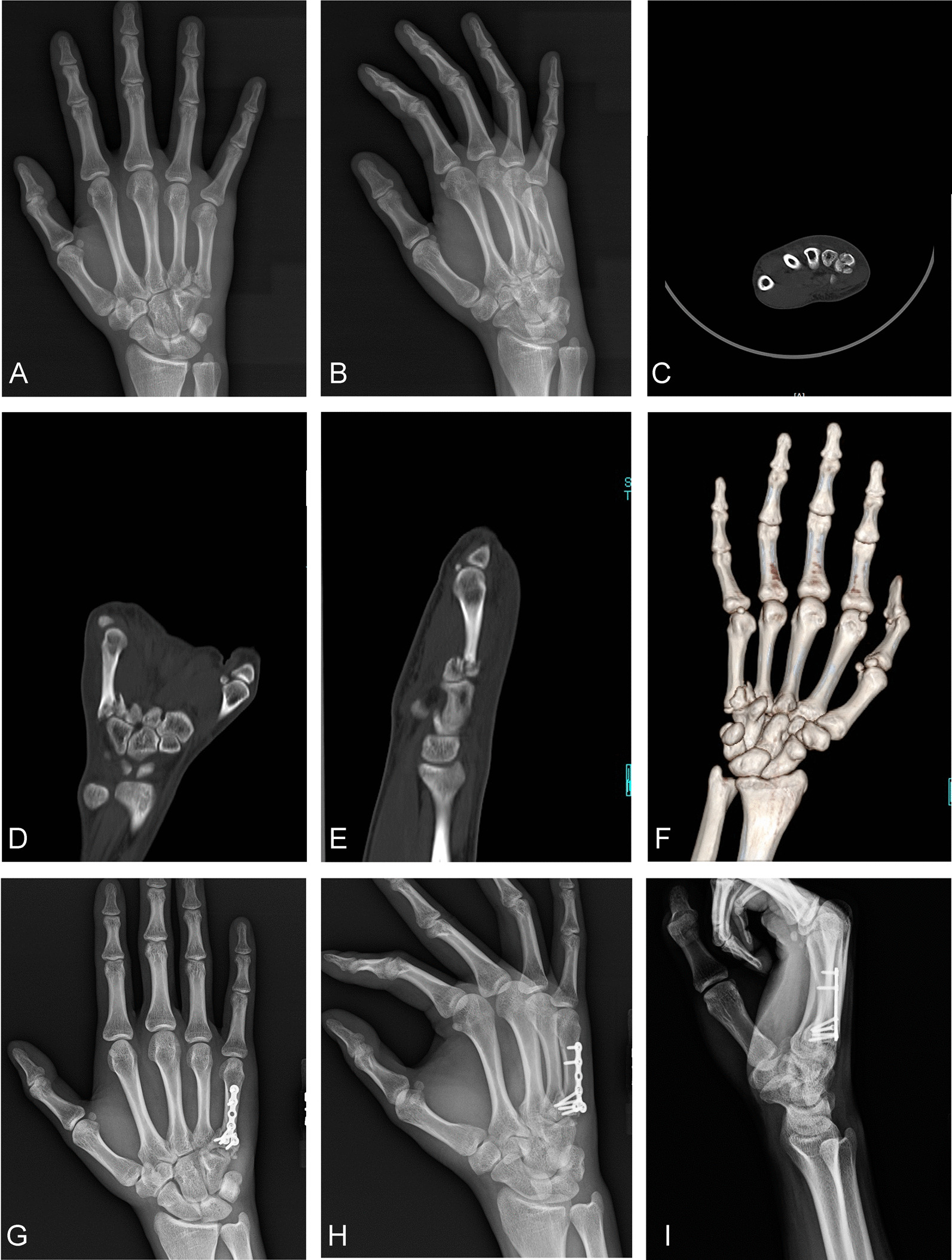
**A**, **B** The preoperative posteroanterior and lateral oblique views of the affected hand on radiograph indicate that there are comminuted fractures of the base of the fifth metacarpal bone, complicated with dislocation. **C**, **D**, **E**, and **F** Preoperative computed tomography and three-dimensional reconstruction of the affected hand show a comminuted fracture of the base of the fifth metacarpal bone that is complicated with dislocation. **G**, **H**, and **I** One-month postoperative posteroanterior and lateral oblique views on radiograph show that the steel plate was not fixed across the joint, and one screw was used to fix the broken fracture block

The group B comprised 50 patients, including 45 males and 5 females with an average age of 28.9 ± 5.7 y (18–56 y). The injuries in group B included fracture of the fourth metacarpal base with dislocation or subluxation (n = 6), fracture of the fifth metacarpal base with dislocation or subluxation (n = 23), and fracture of the fourth and fifth metacarpal base with dislocation or subluxation (n = 21); 46 cases of injury were to the right hand and 4 cases involved injury to the left hand. The injuries were caused by falling (n = 9), boxing (n = 36), and traffic accident (n = 5). The OTA fracture classifications [9] were B1 (n = 28) and C1 (n = 22), and all patients were complicated with dislocation or subluxation. All the injuries were closed fractures, including 7 cases complicated with a hamate fracture. In all patients, the posteroanterior and lateral oblique views of the hand were examined by radiograph and CT preoperatively. The typical images taken during surgery in group B are shown in Figs. 3 and 4.

The study has been approved by the Ethics Committee of Beijing Shunyi District Hospital, and the approval document number is 2022-L-013. After all patients in this study signed the informed consent form, the patient's information was then sealed in a file bag and sent to the operating room, where the surgeon opened the sealed information.

### Surgical procedures

Both groups A and B were treated with brachial plexus anesthesia, A tourniquet was applied to the upper arm on the affected side. An S-shaped incision was made on the dorsal side of the fourth and fifth metacarpal bones on the back of the hand. The skin and subcutaneous tissue were cut while protecting the dorsal branch of the ulnar nerve. The extensor digitorum tendon was pulled to either side to completely expose the basal fracture ends of the fourth and fifth metacarpal bones and the dorsal side of the hamate. Extravasated blood was removed, and the operative field was washed with sterile saline.

Group A: The surgical assistant longitudinally tractioned the bone to reduce the fracture. For comminuted fractures involving the articular surface, the articular surface was fully aligned. If a hamate fracture was also present, reduction of the hamate was completed at the same time; a temporary fixation was made using a Kirschner wire; a 1.5 or 2.0 mm straight, T-shaped, or L-shaped micro-locking plate was placed on the hamate; holes were drilled in sequence, and the screws were affixed. Where possible, the hamate bone fracture was fixed using a steel plate (see Fig. 1D–F), and two screws were implanted on the dorsal plate of the hamate bone. After removing the Kirschner wire, the comminuted fracture block at the base of the fourth and fifth metacarpal bones was checked for stability. If it was unstable, screws or an auxiliary Kirschner wire was used for fixation according to the size of the fracture block (see Fig. 2F–H).

Group B: If a hamate bone fracture was also present, it was exposed at the same time. Screw fixation or Kirschner wire fixation was made according to the location of the hamate bone fracture. In most cases, it was fixed separately with two screws (see Fig. 3F–H). The surgical assistant longitudinally tractioned the bone to reduce the fracture. For comminuted fractures involving the articular surface, the articular surface was fully aligned. A temporary fixation was then made using a Kirschner wire, a T-shaped or L-shaped micro-steel plate (1.5 or 2.0 mm) was aligned with the base of the fourth and fifth metacarpal bones, holes were drilled in sequence, and the screws were inserted. Wherever possible, the fragmented fracture blocks were fixed using screws (see Fig. 4G–I). After removing the Kirschner wire, the comminuted fracture blocks at the base of the fourth and fifth metacarpal bones were checked for stability. If the fracture blocks were small and could not be fixed using screws, Kirschner wire was used for auxiliary fixation. A 4–0 absorbable suture was used to repair the carpometacarpal joint capsule and dorsal ligament. The stability of the carpometacarpal joint was checked for re-dislocation. If it was unstable, Kirschner wire was used to assist in fixing the joint.

During both groups of operation, the fracture reduction, the position of the steel plate, and the length of the screw were examined using an radiograph. Finally, the incision was closed layer-by-layer, and a drainage strip was placed in the wound.

### Postoperative care

The carpometacarpal joint was fixed with braces in both groups to maintain the functional position of the joint, but the metacarpophalangeal joints were not fixed, and active flexion and extension of the metacarpophalangeal and interphalangeal joints were practiced immediately postoperatively. The dressing was changed every three days, and the stitches were removed 2 weeks after surgery. After brace fixation for 3 weeks, movement of the carpometacarpal joint was gradually practiced, and rehabilitation exercises were practiced under guidance. After 6 months of fracture healing, all internal fixation devices were removed in group A. In group B, the internal fixation devices were removed according to the patient's requirements.

### Postoperative follow-up and curative efficacy evaluation

All patients were followed up at the hospital at 2, 4, and 8 weeks and 3, 6, and 12 months postoperatively. If a patient was unwell, he/she visited a doctor at any time without restriction. The reexamination included a clinical physical examination and imaging examination. The imaging examination included anteroposterior and lateral radiographs of the wrist joint and 30-degree pronation oblique radiographs to observe the healing of the fractures. Observe and record the patient's Hand function evaluation during the follow-up period, including handgrip strength, total active motion (TAM) of ring finger and little fingers, wrist pain, and range of motion. The handgrip strength test was conducted using a CAMRY electronic grip tester, repeated 3 times, with the maximum value taken each time. At 3, 6 and 12 months after operation, DASH (disabilities of the arm, shoulder and hand), visual analogue scale (VAS), Cooney' modified Mayo wrist score were used for scoring and comparison to evaluate the clinical efficacy evaluation of patients (Table 1).

**Table 1:** The detail of Cooney’ modified Mayo wrist score

	Scoring
1. Pain (0–25 points)	
Painless	25
Mild or occasional pain	20
Moderate pain but tolerable	15
Severe pain and intolerable	0
2. Functional status (0–25 points)	
Return to normal work	25
Can do limited work	20
Can move but unable to work	15
Immobility due to pain	0
3. Range of activity (Contrast with healthy side 0–25 points)	
100%	25
75–99%	20
50–74%	10
25–49%	5
0–24%	0
4. Power of gripping (Contrast with healthy side 0–25 points)	
100%	25
75–100%	20
50–75%	10
25–50%	5
0–25%	0

Excellent, 90–100 points; good, 80–89 points; normal, 65–79 points; poor, 65 points below

### Statistical methods

Data processing and analysis were performed using the SPSS Statistics 25.0 software package. The measurement data were expressed by mean ± standard deviation, the comparison between the two groups was performed by *t*-test, and the counting data was performed by chi square test. *P* < 0.05 was statistically significant.

## Results

### General results

There were no significant differences in gender, age, fracture type, or other general data between groups A and B (*P* > 0.05; see Table 2). Between group A and group B, there were no differences in the operation time (65.8 ± 14.3 and 66.2 ± 15.5 min, respectively; *P* > 0.05), fracture healing time (70.6 ± 6.6 and 68.8 ± 5.8 days, respectively; *P* > 0.05), or follow-up duration (12.7 ± 2.2 and 12.8 ± 2.1 months, respectively; *P* > 0.05; see Table 3). All the wounds healed after the first intervention, and no wound infection occurred. In group A, the internal fixation devices were removed from all patients 6 months postoperatively. In group B, the internal fixation devices were removed from five patients according to the patients' requirements. The steel plate broke in five subjects in group A (see Fig. 1E, F) and no subjects in group B. No re-dislocation occurred in either group. There were no injuries to the superficial or deep branches of the ulnar nerve in either group. In three patients in each group, a Kirschner wire was used for auxiliary fixation and was pulled out 3 weeks postoperatively with no complications (e.g., pin track infection or Kirschner wire breakage).

**Table 2:** Comparison of preoperative general data between the transarticular fixation group (group A) and non transarticular fixation group (group B) (n, $\overline{x} \pm s$)

	Group A	Group B	X^2^/t values	*P*-value
Gender				
Male	44	45	0.102	0.749
Female	6	5		
Age (years)	28.8 ± 6.1	28.9 ± 5.7	− 0.067	0.946
Fracture type				
B1	29	28	0.041	0.840
C1	21	22		
Whether with hamate fracture				
Yes	9	7	0.298	0.585
No	41	43

**Table 3:** Comparative analysis of operation time, fracture healing time and follow-up time between the transarticular fixation group (group A) and non transarticular fixation group (n, $\overline{x} \pm s$)

	Operation duration (min)	Fracture healing time (d)	Follow-up duration (month)
Group A	65.8 ± 14.3	70.6 ± 6.6	12.7 ± 2.2
Group B	66.2 ± 15.5	68.8 ± 5.9	12.8 ± 2.1
t-value	− 0.120	1.422	− 0.139
*P*-value	0.904	0.158	0.890

### Hand function evaluation

At the 3-month follow-up, in group A and B, the handgrip strength was 31.6 ± 3.6 and 31.6 ± 3.7, respectively (*P* > 0.05), the TAM of the ring finger was 251.6 ± 7.9 and 251.5 ± 7.2, respectively (*P* > 0.05), and the TAM of the little finger was 255.3 ± 9.7 and 255.1 ± 9.1, respectively (*P* > 0.05).

At the 6-month follow-up, in group A and B, the handgrip strength was 40.2 ± 3.1 and 41.4 ± 3.1, respectively (*P* < 0.05), the TAM of the ring finger was 276.6 ± 4.5 and 278.5 ± 4.6, respectively (*P* < 0.05), and the TAM of the little finger was 267.7 ± 8.3 and 270.7 ± 5.3, respectively (*P* < 0.05).

At the 12-month follow-up, in group A and B, the handgrip strength was 42.5 ± 3.2 and 42.0 ± 3.2, respectively (*P* > 0.05), the TAM of the ring finger was 280.4 ± 4.5 and 280.5 ± 4.4, respectively (*P* > 0.05), and the TAM of the little finger was 271.1 ± 4.9 and 271.3 ± 5.8, respectively (*P* > 0.05; see Table 4).

**Table 4:** Grip strength and TAM of ring and little fingers in the transarticular fixation group (group A) and non transarticular fixation group (group B)

	Group A	Group B	*t*-value	*P*-value
Grip strength (kg)				
3 months	31.6 ± 3.6	31.6 ± 3.7	− 0.097	0.923
6 months	41.4 ± 3.1	40.2 ± 3.1	2.006	0.048
12 months	42.5 ± 3.2	42.0 ± 2.8	0.769	0.444
Ring finger TAM (°)				
3 months	251.6 ± 7.9	251.5 ± 7.2	0.053	0.958
6 months	278.5 ± 4.6	276.6 ± 4.5	2.067	0.041
12 months	280.4 ± 4.5	280.5 ± 4.4	− 0.056	0.955
Little finger TAM (°)				
3 months	255.3 ± 9.7	255.1 ± 9.1	0.085	0.913
6 months	270.7 ± 5.3	267.7 ± 8.3	2.139	0.035
12 months	271.1 ± 4.9	271.3 ± 5.7	− 0.103	0.918

### Clinical efficacy evaluation

At the 3-month follow-up, in group A and B, the DASH scores were 7.0 ± 4.0 and 7.1 ± 4.4, respectively (*P* > 0.05), the VAS scores were 1.1 ± 0.9 and 1.2 ± 1.0, respectively (*P* > 0.05), and the Mayo scores were 86.0 ± 10.3 and 85.1 ± 11.1, respectively (*P* > 0.05).

At the 6-month follow-up, in group A and B, the DASH scores were 2.1 ± 2.7 and 3.3 ± 3.0, respectively (*P* < 0.05), the VAS scores were 0.8 ± 0.9 and 1.2 ± 1.0, respectively (*P* > 0.05), and the Mayo scores were 92.6 ± 7.8 and 88.6 ± 10.0, respectively (*P* < 0.05).

At the 12-month follow-up, in group A and B, the DASH scores were 1.9 ± 2.6 and 1.9 ± 2.4, respectively (*P* > 0.05), the VAS scores were 0.6 ± 0.7 and 0.6 ± 0.8, respectively (*P* > 0.05), and the Mayo scores were 93.8 ± 8.0 and 93.2 ± 7.5, respectively (*P* > 0.05; see Table 5).

**Table 5:** DASH score, VAS score and Cooney’ modified Mayo wrist score of the transarticular fixation group (group A) and non transarticular fixation group (group B)

	Group A	Group B	t-value	*P*-value
DASH score				
3 months	7.0 ± 4.0	7.1 ± 4.4	−0.166	0.868
6 months	2.1 ± 2.7	3.3 ± 3.0	−2.092	0.039
12 months	1.9 ± 2.6	1.9 ± 2.4	0.040	0.968
VAS score				
3 months	1.1 ± 0.9	1.2 ± 1.0	−0.738	0.463
6 months	0.8 ± 0.9	1.2 ± 1.0	−2.061	0.042
12 months	0.6 ± 0.7	0.6 ± 0.8	−0.134	0.893
Cooney’ modified Mayo wrist score				
3 months	86.0 ± 10.3	85.1 ± 11.1	0.421	0.675
6 months	92.6 ± 7.6	88.6 ± 10.0	2.274	0.025
12 months	93.8 ± 8.0	93.2 ± 7.5	0.388	0.699

## Discussion

The fourth and fifth carpometacarpal joints of the hand are two atypical saddle joints comprising the bases of the fourth and fifth metacarpal bones and the distal end of the hamate [10, 11]. The fourth and fifth carpometacarpal joints are micro-motion joints that participate in the composition of the transverse and longitudinal arches of the hand and are involved in the object holding function of the hand through conical motion [12]. Clinically, the hamate and the metacarpal joint are often regarded together as a unit with many combinations, but at present, there is no systematic and unified classification of these various combinations [13–15]. The main clinical signs are local swelling, tenderness, and a lack of grip strength. As a result, the deformity caused by the fracture and dislocation is not obvious. For patients with an unclear diagnosis or whose carpometacarpal joint must be evaluated, CT and three-dimensional reconstruction should be used [16, 17].

Through radiograph examination, this study revealed that the basal fracture of the fourth and fifth metacarpal bones of the hand may be complicated by dislocation, subluxation, or even fracture of the hamate. According to OTA fracture classification [9], fourth and fifth metacarpal basal fractures can be types A1, B1, or C1. Type A1 is an extra-articular fracture, and its treatment is relatively simple. Types B1 and C1 are complicated with dislocation or subluxation, and the treatment is more complex. Although the reduction may be simple, it is difficult to maintain the reduction as the fracture is unstable. Conservative plaster fixation is suitable for most patients with minor non-displaced fractures, but this approach can cause joint stiffness, malunion, and pain in patients with intra-articular fractures with dislocation or subluxation [18], which can have a serious effect on hand movement. Kirschner wire fixation [19] is often used clinically, particularly for simple dislocation or subluxation of the carpometacarpal joint. The fixation of a fracture and dislocation using a Kirschner wire is a simple procedure, causes minimal trauma, and has a low cost. However, in cases of a comminuted fracture, Kirschner wire fixation has several disadvantages, e.g., insufficient fixation strength [20], it affects tendon sliding, it can cause troublesome postoperative needle tract nursing, and is unfavorable to resuming activities early [21]. Internal fixation with a microplate can achieve anatomical reduction and strong fixation, and early functional exercises can be carried out postoperatively to restore hand function as quickly as possible [22]. There is currently no unified agreement that a microplate should be used for direct fixation or transarticular fixation of a fracture.

Berg and Murph [23] revealed that the ligaments between the fourth and fifth metacarpal bones were strong and stable. If the interosseous ligament is not damaged, even if a dislocation occurs at the base of either the fourth or the fifth metacarpal bones, the other fracture will not be dislocated. If the fracture is complicated with a hamate fracture, this can be fixed using only one or two screws, and if the carpometacarpal joint is still unstable, Kirschner wire fixation can be used.

This clinical comparative study revealed that there were no significant differences in surgery time and fracture healing time between the two groups, suggesting that the different operations are equally difficult. In addition, there was no significant difference in the healing of the fracture or dislocation between the two groups. However, the range of motion and grip strength of the carpometacarpal joint were different between the two groups at certain times. In the first 3 months postoperatively, there was no difference in the range of motion or grip strength. This may have been due to several reasons. Physically, the surgical scar had not yet completely softened, and tendon adhesion and sliding were limited. Psychologically, the patients were reluctant to move and exert themselves for fear of causing local discomfort or a recurrence of the fracture or dislocation. Therefore, differences between the groups in terms of the range of motion and grip strength were not obvious. After 6 months, the fractures had healed, and there was no pain or discomfort in the area. This, coupled with gradual recovery exercises, revealed a difference between the groups. In transarticular fixation, it was not possible to move the fourth and fifth carpometacarpal joints of the hand, and the hand could not hold objects effectively using conical movement. This resulted in a reduction in the range of motion and grip strength. All the internal fixation devices were removed from the patients in group A at 6 months, and some of the internal fixation devices were removed from the patients in group B. Prior to the removal of the devices, there were no significant differences in the range of motion and grip strength of the carpometacarpal joints between the two groups. However, after removing the internal fixation devices, the carpometacarpal joints of the patients in group A were no longer limited, and the function of the carpometacarpal joint had been restored. In group B, the removal of the internal fixation devices did not affect the mobility of the carpometacarpal joint.

During follow-up, it was noted that, during rehabilitation exercises, some patients in group A broke the micro-steel plates. Therefore, rehabilitation exercises should not be too vigorous before the fracture has healed. If the steel plate breaks before the fracture has healed, the steel plate will become ineffective, resulting in displacement, nonunion, or malunion of the fracture.

There were no injuries to the superficial or deep branches of the ulnar nerve in either group. Therefore, sufficient protection was given to the superficial branches of the ulnar nerve under direct observation during the surgery. In addition, a locking plate was employed in this study, and when drilling and screwing, the surgeon only passed through one cortex (not the contralateral cortex), which minimized the damage to the deep branches of the ulnar nerve. There was no statistically significant difference in the postoperative VAS pain scores between the two groups at any time. The reason for this may have been because there was no material difference in the degree of soft tissue injury during the surgery. The differences in the DASH and Mayo scores were due to joint fixation and loss of movement.

Some scholars used Kirschner wires to fix basal fractures and dislocations of the 4th and 5th metacarpal bones, such as Valente et al. [24] who believed that closed reduction and percutaneous Kirschner wire internal fixation technique was the most effective method for treating basal fractures and dislocations of the 4th and 5th metacarpal bones; Schortinghuis et al. [7] believed that using open reduction and internal fixation technique to treat unstable 4th and 5th metacarpal basal fractures and dislocations could also achieve good results. We believe that the open reduction and internal fixation technique was more difficult than the Kirschner wire fixation technique and requires experienced physicians to perform the operation.

The advantages of direct internal fixation with a microplate are as follows: For intra-articular fractures at the base of the fourth and fifth metacarpal bones, it is possible to completely align the articular surface, place steel plates, and implant screws to ensure firm fixation of the fracture. Even in comminuted metacarpal basal fractures with fracture blocks on the volar or lateral sides of the base connected with ligaments and articular capsules, when the bone structure was restored, the dislocation of the joint became stable. In addition, the dorsal ligament and articular capsule must be repaired after the fracture repair to ensure that dislocation does not recur. During functional exercises and long-term activities, the steel plate is not placed under stress and, as such, will not break. In addition, the microplate can be removed without a second surgical procedure. The disadvantages of direct internal fixation with a microplate are as follows: When the base has a comminuted fracture and the fracture block is very small, it is difficult to implant the screw, and a Kirschner wire will be required to assist in fixation. Cases with a hamate fracture require a separate screw or Kirschner wire fixation, which makes the operation cumbersome. If the fracture is a small avulsion fracture with carpometacarpal dislocation, it cannot be directly fixed using a microplate.

The advantages of transarticular fixation with a microplate are as follows: For an intra-articular fracture at the base of the fourth and fifth metacarpal bones, after reduction of the dislocation or subluxation, the micro-steel plate directly crosses the joint and applies pressure to the fracture site; as such, there is no possibility of re-dislocation. If the fracture is complicated with a hamate fracture, the fractures can be fixed together. When the dorsal bone cortex is intact and there is a fracture block on the volar side, the volar fracture block cannot be observed using the dorsal surgical approach. After traction reduction, the steel plate is placed across the dorsal side of the joint, applying direct pressure, and the screw is implanted. The operation is simple and fast. Under the support of the trans-carpometacarpal joint steel plate, the joint space can be effectively extended to reduce the occurrence of joint stiffness [25], and even small avulsion fractures with dislocation or subluxation of the carpometacarpal joint can be treated with microplate transarticular fixation.

The disadvantages of transarticular fixation with a microplate are as follows: The scope of surgical stripping is slightly larger than direct internal fixation, making it better suited to cases that are complicated by a hamate fracture. The currently available types of micro steel plates are T-shaped or L-shaped, and when two screws at the one end of the T-shaped or L-shaped types are affixed to the hamate, only one screw can be affixed to the fracture site. If the base of the fourth and fifth metacarpal bones has a comminuted fracture of the articular surface, it will be difficult to control the fracture alignment with one only screw; accordingly, additional support by auxiliary screws will be needed. If the fracture block is small, a Kirschner wire will also be required for auxiliary fixation, which will flatten the articular surface [26] and reduce the occurrence of osteoarthritis. The microplate for transarticular fixation must be removed during a second surgery within a given timeframe after the fracture heals, otherwise, the lack of movement in the carpometacarpal joint over a prolonged period can result in arthritis, pain, stiffness, or loss of function in part of the hand.

## Conclusion

In summary, for a basal fracture of the fourth and/or fifth metacarpal bones of the hand with dislocation or subluxation, both microplate direct and transarticular fixation can achieve fracture fixation and healing. Both of these methods presents advantages and disadvantages. The specific method to be used in clinical practice needs to be selected according to the surgeon's understanding, the operation methods he is good at and the degree and type of fracture dislocation.

## Data Availability

All data generated or analysed during this study are included in this article. Further enquiries can be directed to the corresponding author.

## Abbreviations

OTA: Orthopedic Trauma Association
TAM: Total active motion
CT: Computed tomography
